# Automated entrance monitoring to investigate honey bee foraging trips using open-source wireless platform and fiducial tags

**DOI:** 10.1016/j.ohx.2024.e00609

**Published:** 2024-11-19

**Authors:** Diego Penaloza-Aponte, Sarabeth Brandt, Erin Dent, Robyn M. Underwood, Benedict DeMoras, Selina Bruckner, Margarita M. López-Uribe, Julio V. Urbina

**Affiliations:** a School of Electrical Engineering and Computer Science, The Pennsylvania State University, University Park, 16802, PA, USA; b Department of Geography, Texas A&M University, College Station, 77843, TX, USA; c Department of Entomology, The Pennsylvania State University, University Park, 16802, PA, USA; d Department of Entomology, Cornell University, Ithaca, NY 14853, USA

**Keywords:** Automated image monitoring, Field instrument, Insects behavior, Raspberry Pi, Fiducial tags

## Abstract

Honey bee foraging is a complex behavior because it involves tens of thousands of organisms making decisions about where to collect pollen and nectar based on the quality of resources and the distance to flowers. Studying this aspect of their biology is possible through direct observations but the large number of individuals involved in this behavior makes the implementation of technologies ideal to scale up this type of study. Consequently, there is a need for instruments that can facilitate accurate assessments of honey bee foraging at the colony level. To address this need, this work aimed to develop an automated imaging system for monitoring the in-and-out activity of honey bee foragers as they walk through a customized entrance with a camera sensor at the hive entrance. We used AprilTags attached to each bee’s thorax to provide unique identification numbers that allowed the system to track in-and-out events throughout the foraging season of the colony. Our design relies on low-cost Raspberry Pi computers and cameras, along with commercially off-the-shelf components, making it easily reproducible with the open-source documentation provided. We successfully deployed and evaluated our system in six locations, demonstrating consistent results. In this paper, we present the details about the development of the system, data collected from multiple colonies, and post-processing analysis from one of our apiaries. Our results highlight the system’s effectiveness in monitoring honey bee trips, capturing various behaviors associate with their activities outside the colony, which lay the groundwork for future estimations of foraging distances.

## Graphical abstract

**Figure:**
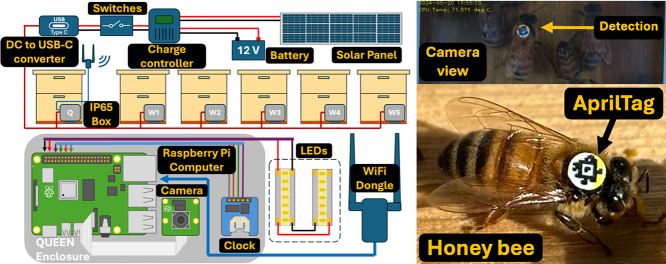

**Table:** 

**Specifications table**	

**Hardware name**	*BeeCam-AprilTag*
**Subject area**	•*Engineering and material science*
	•*Biological sciences*
	•*Environmental, planetary and agricultural sciences*

**Hardware type**	•*Imaging tools*
	•*Field measurements and sensors*
	•*Electrical engineering and computer science*

**Closest commercial analog**	*No commercial analog is available.*
**Open source license**	Creative Commons Attribution 4.0 International
**Cost of hardware**	<*$1500 USD*
Source file repository	https://doi.org/10.5281/zenodo.13227905

## 1 Hardware in context

*Apis mellifera*, commonly known as the western honey bee, is the most important managed pollinator for agricultural crops and the production of goods such as honey, wax, and propolis. Furthermore, products derived from honey bees serve as valuable bioindicators of environmental pollutants, such as heavy metals, pesticides, and airborne particles [1]. In recent decades, concerns over food security due to declines in the number of honey bee colonies have increased [2], [3]. Despite the multifactorial nature of the stressors that affect bee pollinators, one of the most important factors associated with honey bee health is the quality of the landscape surrounding the colony [4], [5]. Thus, characterizing the foraging activity of bees under different conditions offers a powerful tool to understand questions, such as how much they utilize floral resources in different landscapes and how their foraging activity changes based on the presence of stressors such as pesticides. However, because of the large size of honey bee colonies (>20,000 individuals) and the long activity period of the colony (all year long), studying foraging behavior through direct observations is generally a time-consuming endeavor. Thus, there is a critical need to develop automated tools that can be used to accurately assess honey bees’ foraging activities at the colony level. In recent decades, numerous automated systems have been implemented to monitor bee behavior [6], pests [7], weather conditions [8], [9], and flight activity outside colonies [10], [11] using various technologies and techniques. Harmonic radar technology, for example, offers a way to spatially track foragers over large geographic areas, allowing the monitoring of bee trajectories in the landscape. However, this technology requires attaching bulky transponders to the bees, which can affect their flight ability, and it only allows for tracking individual targets rather than multiple bees on a large scale [12]. On the other hand, radio-frequency identification (RFID) technology has emerged as a solution for monitoring in-and-out activities within a colony, providing identification for each individual bee. However, the effects of heavy tags and strong electromagnetic waves produced by RFID readers on honey bee behavior are still unknown. More importantly, for large-scale tagging experiments where thousands of tags are required, cost and the limited number of available tags can be significant limiting factors to study honey bee foraging behavior [13]. To eliminate the need for tagging each bee, a system based on infrared sensors was developed to detect in-and-out activity [14]. A customized passageway was built at the hive entrance with multiple infrared sensors to count incoming and outgoing events. However, since the system lacks a way to identify each individual bee with their corresponding event, it is not possible to estimate trip duration based on individual data. Similarly, a system using image processing techniques was developed to count activity at the hive entrance [15]. Both systems consist of multiple devices connected in a network, capable of uploading data to the cloud using 4G LTE (Fourth Generation Long-Term Evolution) routers. Comparing the methods described earlier, image processing has the potential to provide more information about bees like: body shape, attached pests, behavior, trajectories, etc, demanding further detection and analysis. These tasks can consume considerable computing resources, often requiring computers with graphic processing units (GPU), which could demand even more power.

Despite the hardware and processing challenges associated with image-based systems, a technique using two dimensional (2-D) barcoded paper tags has increased in popularity to monitor and identify bees in a colony because of the relative low cost and simplicity in tags production [16], [17], [18]. These types of systems rely on cameras to detect and extract information from tags carried by the bees. In particular, the use of “fiducial” tags, has addressed the need for expensive computing, since these types of 2-D barcoded tags have a very small payload, and can be detected efficiently in cluttered images using fewer computing resources. Based on this method, an image system to monitor foraging trips of individual bees was successfully implemented using infrared cameras along with circular character-encoded tags [19]. The experimental results presented demonstrated that this image-based technique is feasible and capable of determining the foraging trip duration of individual bees.

Numerous open-source fiducial tag families and detectors have been developed and optimized to improve detection speed, reduce false alarm rates, add more tag shapes, and enhance tag localization and orientation, all while consuming fewer computing resources. Fiducial tags have been widely used in various applications such as, augmented reality, robotics, camera calibration and even specifically for tracking bees as demonstrated in [20], which is a tag family based on ARtag [21] and CALtag [22]. Among the many tag families available in the literature, we focus on the use of AprilTags [23] due to their comprehensive documentation, active repository, and support for both C and Python implementations. Systems using AprilTags have been implemented for different tasks including the study of honey bee behavior in small observation windows [24]; the trajectory augmentation of automatic honey bee monitoring [25]; and the identification of foraging trips of bumble bees [26]. The latter application is of particular interest since it demonstrates the feasibility of a system that relies on low-cost hardware and open-source tools, without the need for expensive high-resolution cameras and high-performance computers.

Here, we present the development of the BeeCam-AprilTag system that monitors the in-and-out activity of honey bee individuals in a hive and allows for the estimation of honey bee trip duration. We provide full documentation with instructions on how to reproduce and operate our system, insights into the implementation process, the challenges encountered, and some limitations. In addition, we present preliminary results from data collected during the spring-summer season of 2024, showcasing the post-processing analysis performed by the system. We demonstrate that our technique is feasible and capable of determining the trip duration of hundreds of individual bees on a daily basis. Both software and hardware were programmed in Python, and use the latest version of AprilTag3 [23], which offers high performance, more flexibility, and more features than previous generations of these tags [20], [21], [22]. The other advantage of this system is that it is based on low-cost hardware and open-source software that allows for the implementation of this system for other applications. Additionally, our design can be fully implemented in remote field areas as it is portable, has power autonomy, is weather resistant, and is relatively low cost. Most components are commercially off-the-shelf (COTS), making them easy to access and replace. The core of the system is based on Raspberry Pi (RPi) single-board computers and cameras, ensuring low-cost computing processing and the use of open-source software.

## 2 Hardware description

The BeeCam-AprilTag system was created using four objectives:

• Integrate a real-time detection system capable of identifying individuals and retrieving their position and orientation using inexpensive tags.
• Create a system composed of multiple devices (at least six) able to collect and share data in a local wireless network that users can access wirelessly for monitoring and data transfer.
• Assemble a portable system, deployable outdoors for long periods (several months), and built entirely with commercially available off-the-shelf (COTS) components accessible and fast to replace.
• Design an architecture and interface that is simple to install and operate by people with limited knowledge in electronics and programming.

Based on these attributes, a system design was implemented, and its fundamental features are described in the following four sections:

### 2.1 Electronic design

**Fig. 1:**
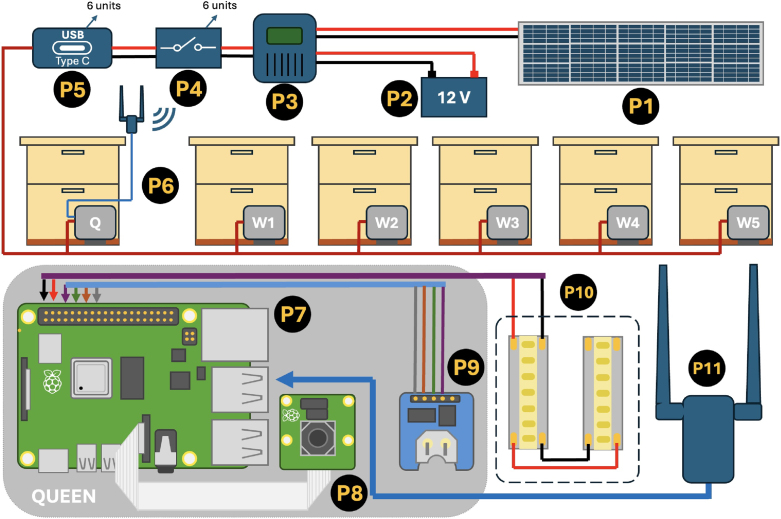
General system schematic describing the main hardware components. Labels P1 to P5 indicate the power module components, label P6 refers to the enclosures containing the electronics for each colony, and labels P7 to P11 denote the electronic components within the enclosure of the “queen” computer. Further details of the labeled parts can be found in Section 3 (Bill of Materials).

The main structure of the system is based on a wireless local network of six Raspberry Pi 4 (RPi-4) single-board computers attached to Raspberry Pi cameras module 3 (RPiC-3), with the addition of extra low-cost electronic components and an autonomous power module. A schematic of the system is depicted in Fig. 1 showing the most important pieces of hardware. The power module consists of a 12 volt (V) Lithium Iron Phosphate (LiFePo) battery with a 100 amper hours (Ah) capacity, which is charged by using a 12 V monocrystalline solar panel of 195 watts (W), in combination with a solar charge controller that uses Maximum Power Point Tracking Techonology (MPPT) to ensure a high conversion efficiency of 98% and fast charging speed. This controller is also a regulator that provides a stable voltage source to the loads equal to the battery voltage. A switch was installed for each RPi-4 along with a waterproof 12–24 V direct current (DC) converter to USB-C male output adapter with a conversion efficiency of 93%. Electrical wire (18 gauge) was used to supply power to each RPi-4 placed in front of the hive entrances, having them spread in apiaries of a maximum of 10 m radius. Fig. 1 shows the electronics installed on each hive entrance. In this case, we can observe the hardware for the “queen” computer, which is named as such because it is the main computer that generates a WiFi Hotspot. The rest of the RPi-4’s are called “worker” computers, which connect to queen’s WiFi Hotspot to transfer the data. Consequently, all computers share the same core hardware composed of: the Raspberry Pi 4 model B with 2 GB RAM, an SD card with 128 GB of storage, a 12 megapixel Raspberry Pi camera module 3 model Wide, and two strips of LEDs powered by the GPIO (General Purpose Input Output) pins. For the “queen”, two extra pieces of hardware were added: the real-time clock and an external WiFi dongle as illustrated in Fig. 1. The real-time clock PCF8523 utilizes SPI (Serial Peripheral Interface) communication protocol to update the time and date of the queen’s RPi in case it loses power, as it lacks an internal clock. Since the “workers” do not possess either an internal or external clock, they synchronize their date and time with the “queen” via the NTP protocol using the “timedatectl” service, which is part of the Raspberry Pi operating system (OS). This synchronization occurs over the local wireless network. Therefore, maintaining a stable and uninterrupted wireless connection across the system is crucial. The RPi’s board possesses an embedded WiFi/Bluetooth card, capable of establishing a WiFi Hotspot that can handle up to five devices connected at the same time. This limited number of devices for connection represents an inconvenience for our application since an external device like a laptop, phone or tablet is used to connect remotely to the “queen” network and monitor all the devices. Note that in previous tests we performed in the field, the RPi internal WiFi card used as a Hotspot also demonstrated a low coverage, less than 4 meter radius, that was often unstable and slow. Later, we found out it was mainly due to the proximity of the computers to the ground, which produced interference with the WiFi signals.

Since the “queen” is the main node that generates the Hotspot WiFi, it requires to establish stable links to each “worker” over an area of 10 m radius at least. Consequently, the PAU0D WiFi dongle, which has external high-gain antennas, was selected to provided a complete reliable coverage to the apiary. This WiFi adapter is a “plug-and-play” dongle, which means it does not require the installation of any driver since it can work with the Linux kernel drivers of the Raspberry Pi OS. This WiFi dongle was installed (only in the “queen” computer) between 1.5 to 1.7 m above ground to avoid ground interference using a USB 3.0 connection to ensure high-speed communication.

We measured the total power consumption of the system, which includes one queen unit and five worker units, considering all the electronics described in Fig. 1 (from components P4 to P11). The system demonstrates an average power consumption of 32 W. Since it operates continuously for 24 h, it consumes 768 watt-hours (Wh) per day. Our current power system setup, with a solar panel capacity of 195 W, is sufficient to keep the battery above 50% capacity during consecutive sunny days. However, in the scenario of two consecutive cloudy days, the battery can become completely depleted. To address this issue, the power system would require a solar panel capacity of at least 768 W to compensate for the power consumption in 24 h during periods of low solar availability, such as very cloudy days. This capacity would ensure a fully autonomous system that can operate 24 h uninterrupted, even in less-than-ideal weather conditions. However, due to budget constraints, increasing the solar capacity was not feasible for our system design. Instead, our beekeeper collaborators, who visit the apiaries weekly for maintenance, also monitor the system and anticipate potential cloudy periods. When necessary, they were dispatched to swap batteries to prevent system shutdown. This proactive approach allowed us to maintain system functionality throughout the entire summer season.

### 2.2 Mechanical design

**Fig. 2:**
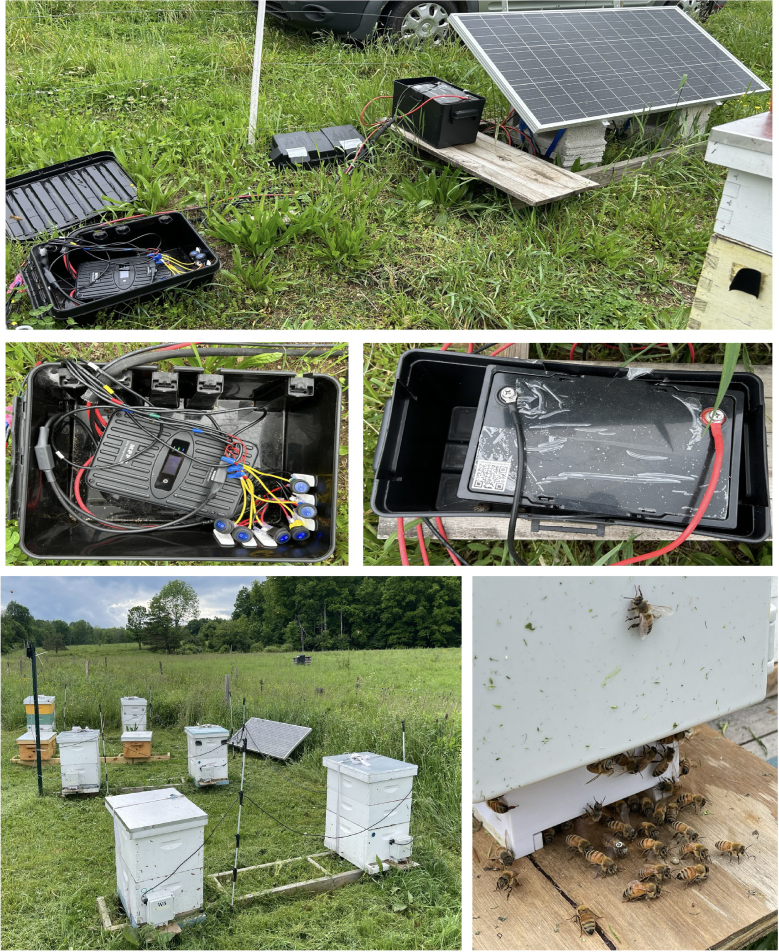
BeeCam-AprilTag system installed in six colonies of an apiary. The top figure shows the power module deployed in the field. The center figures illustrate the solar controller and battery housed inside waterproof enclosures. The bottom figures demonstrate the electronics, with their enclosure, installed at the colony entrances.

The system was designed to be deployed outdoors for several months to continuously collect data in apiaries located at farms. Therefore, it is expected to endure environmental conditions characterized by high humidity, heat, and winds. In consideration of this, waterproof enclosures were selected for hardware such as batteries, controllers, and switches as depicted in Fig. 2. We can observe, two black boxes, containing the hardware mentioned, tightly attached using waterproof wires a few inches above the ground to avoid any water infiltration.

Additionally, IP65 enclosures were chosen for single-board computers and cameras to ensure both water and dust proofing. These enclosures were modified to mount the camera sensor and LED strips at the base of the box and also accurately attach to the 3-D printed entrance for bees. Special considerations and adjustments were taken into account to find the optimal values for parameters like: focal distance, tunnel field of view area, camera resolution, entrance height/width and the available area for structural support. Therefore, a 3-D model using SketchUp software was created to adjust these parameters and develop a final prototype. Original CAD files can be found in our repository (3D-printed-entrace-files.zip) with the supplementary 3-D printing files for the entrance. In our final design, we placed the camera 2 inch away from the translucent glass, which the camera uses to see through the entrance tunnel. This gave us a tunnel field of view of 2 × 3 inches, making the tunnel entrance for the honey bees 2 inch wide by 1/4 inch high. The entrance was designed to fit bolts and wing nuts on the sides to attach the RPi enclosure. The 3-D printed entrance was fabricated using fused deposition modeling (FDM) since it is generally affordable and widely available. Meanwhile, the material chosen was ASA (acrylic styrene acrylonitrile) because it is UV-resistant and suitable for outdoor applications.

**Fig. 3:**
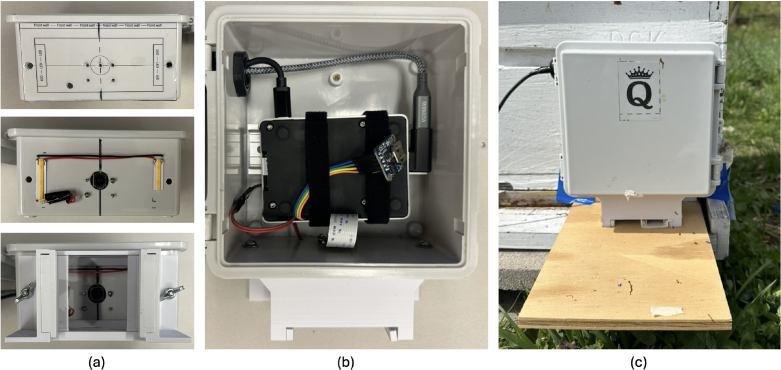
Hardware for queen computer. (a) IP65 enclosure modifications. (b) Electronics inside enclosure. (c) Enclosure, 3-D printed entrance and landing board installed at colony entrance.

For the RPi enclosure, translucent printed stickers were used to accurately drill and install components like cameras, LEDs, and entrances, Fig. 3 shows the enclosure before and after modifications, respectively. CAD files for enclosure modification can be found in our repository with the name Enclosure_modifications.zip.

### 2.3 Fiducial tags

**Fig. 4:**
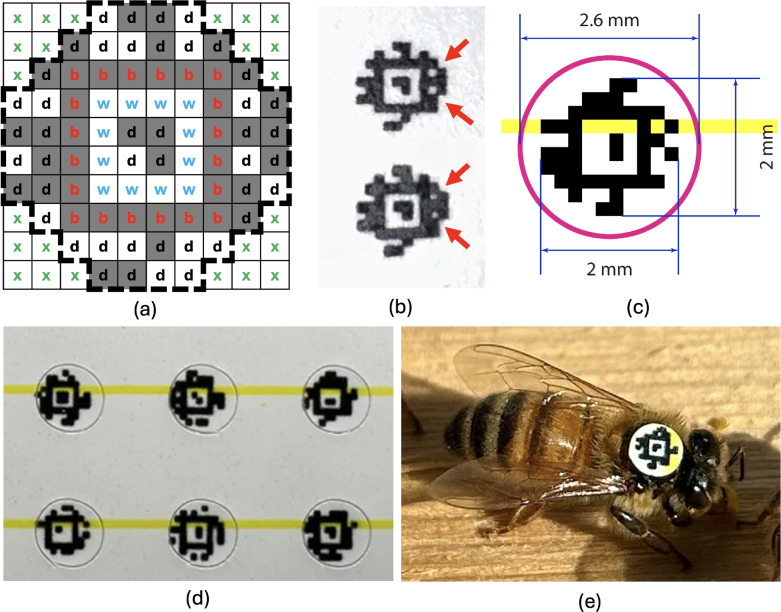
AprilTags: (a) Pixel-bit structure for the 44h12 circular tag family. (b) Printing quality comparison, with arrows indicating white pixels obscured by ink smearing. (c) Printed tag dimensions, showing the magenta circle for the cutting area and the yellow strip for tag orientation. (d) Tags printed and cut, ready for use. (e) Tag mounted on the thorax of a honey bee. (For interpretation of the references to color in this figure legend, the reader is referred to the web version of this article.)

Fiducials are artificial visual markers engineered for quick and easy detection in cluttered images. They resemble 2-D barcoded systems like QR (Quick Response) codes but differ significantly in detectability speed and data payload. Unlike QR codes, which can store hundreds of bytes (1 byte = 8 bits) of data and demand high-resolution and precise alignment for detection, fiducials carry only a few tens of bits. They can be automatically detected even under conditions of low resolution, poor illumination, and random rotation. Furthermore, multiple tags can be detected simultaneously in a cluttered image, providing payload information, as well as the camera-relative position and orientation for each tag [27]. Traditional fiducial tags lack a certain degree of customization in terms of data density, border size and shape, being in most cases standard square layout designs that do not make efficient usage of circular space like the thorax of a bee. AprilTag fiducial tags, however, solve these problems by proposing a flexible tag layout where users can generate a set of data bits arranged in a desired shape with a low false positive rate detection [28]. The available area for tagging is delimited by the honey bee’s thorax, allowing tags with a maximum of 2.6 mm diameter in size that do not obstruct the worker honey bee’s wings or head, avoiding any detriment in their flight ability. In order to tag thousands of bees for an entire spring-summer season, a new Apriltag family with circular shape and enough data bits to fit at least 6000 different ID numbers were required. In this way, the circular tag family 44h12 was generated using the generation code provided by the Apriltag open-source repository [29]. This family provides circular tags that contain 44 data bits with a hamming distance of 12. Generating this type of tags can be an expensive computational operation. While small tags can take few hours, large tags (pixels arrange greater than 6 × 6) can take a few days to compute [27]. In our case, after almost 24 h we generated 8959 tags. More tags could have been generated but we decided to stop the program since we already surpassed the minimum number of tags we required. Fig. 4(a) shows an arrangement 10 × 10 pixels that contains the bit-pixel structure for the 44h12 circular family. Each pixel is labeled with a letter that describes its function in the tag computation. The pixels white (‘w’) and black (‘b’) are fixed to those colors and are used as a border for initial tag detection. Data (‘d’) pixels are used to store information in a binary format where white corresponds to ‘1’ and black color to ‘0’. The pixels near the corners with the letter ‘x’ represent the pixels ignored in computation, thus generating a tag with a raster approximation to a circle. Initially, tags were fabricated using office printers and cut by hand. However, for large-scale production, a cutting plotter machine Graphtec model CE5000-60 was used to cut 6000 tags with high precision. We encountered challenges worth mentioning in the printing and cutting process. The most notorious was that quality printing can considerably affect tag detection. We encountered scenarios where different printers operating with the same settings produced tags with consistently different quality. As shown in Fig. 4(b), two printers were used to create 2 mm×2 mm tags at a resolution of 1200 DPI. The black ink for the tag on the bottom bleeds more and heavily obscures multiple white pixels (indicated by red arrows), while the tag on top has clearer white pixels. Tags with white pixels obscured by ink bleeding are often undetectable. The simplest solution was to avoid printers that produce this effect. However, for scenarios where the problem persists and access to good quality printers is limited, one could implement a solution through software where the white pixels inside the image are digitally dilated before applying the AprilTag detector. This process was tested empirically and proved to be a great solution to bring back the detection of all tags. This solution was implemented in the program “beecam2_preview.py” and can be disabled if necessary by commenting those lines of code. Tags for large scale fabrication were printed with dimensions of 2 mm × 2 mm and a cutting diameter of 2.6 mm as illustrated in 4(c). This gave us room for error in the cutting process since the machine precision was 0.1 mm. Fig. 4(d) shows fabricated tags, ready for installation. Fig. 4(e) illustrates one of the tags correctly mounted on a honey bee worker’s thorax. A yellow line, which does not interfere with detection, was added to help users identify the direction tags need to be installed. The yellow line is placed on the “forward” facing edge of the tag, toward the head of the honey bee. The tags were printed on matte sticker paper to secure the tags while they were machine cut. Finally, tags were coated with at least 4 thin layers of acrylic sealer with a matte finish to protect them from getting scratched or smeared by any liquid. Matte finish for tags is important to avoid any glare that can affect their detectability. For all of our experiments, paper tags were attached to bees using natural glue (shellac) since it induces less damage to the flight muscles compared to synthetic glue (cyanoacrylate), allowing the study of bees for longer periods [30]. Tag family 44h12 ready for printing and cutting can be found in our repository, in the file AprilTags_files.zip. We have also included detailed instructions on how to tag bees, along with a list of necessary materials, in the document titled “BeeCam_AprilTag_Manual.dox”.

### 2.4 Interface and operation

**Fig. 5:**
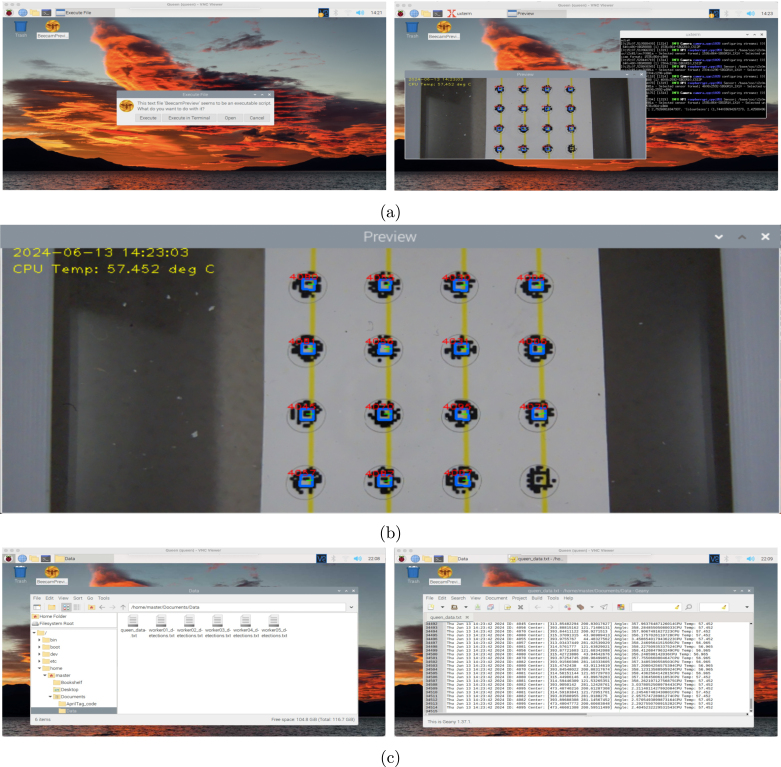
BeecamPreview demonstration. (a) BeecamPreview program manual execution. (b) BeecamPreview detecting AprilTags in real time. (c) Data stored by BeecamPreview. (For interpretation of the references to color in this figure legend, the reader is referred to the web version of this article.)

The system can be accessed through the Hotspot WiFi created by the “queen” computer using RealVNC Viewer on any device, like a laptop or tablet. RealVNC Viewer does not require an account to use the software in a local network and provides a stable graphical remote access interface. The queen possesses a static IP address in the same way as the workers. Therefore, once the queen boots up, its WiFi Hotspot is automatically available, allowing the workers to connect to the network and update their time and date settings accordingly. All devices execute our program beecam2_preview.py automatically on boot. This program applies the AprilTag detector in real-time to each frame captured by the camera. To speed up the process of detection, multiple threads in the CPU are initialized to run the detector over multiple frames in parallel. This results in a system able to process up to 15 frames per second, depending on the amount of clutter in the images. After a tag has been detected, the program stores its information (ID, orientation, camera-relative position), and a date/time stamp is added, along with the CPU temperature at the time of detection (to help us monitor computer status). This information is stored in a text file format, with one detection per line as illustrated in Fig. 5(c). Every minute, the workers transfer copies of their data text files to a shared folder located in the queen computer. This setup enables users to retrieve the entire system’s data through a single connection to the queen. In case our program needs to be run manually, an executable was created on the desktop, as depicted in Fig. 5(a). Once the program is executed, a preview of the camera is displayed, showing the tunnel entrance as illustrated in Fig. 5(b). The program detects tags in real time, drawing a blue square around the tag border with a green bar indicating the forward orientation of the tag, along with the ID number printed in red. When the blue frame is seen on top of the tags, the text file is updated with that detection. This visual feedback assists the user in testing the system and ensuring its functionality.

## 3 Design files

*Design files summary table*

**Table:** 

Design filename	File type	Open source license	Location of the file
Bill_of_Materials	Xlsx	CC BY 4.0	https://doi.org/10.5281/zenodo.13227905
3D-printed-entrance_files	Zip	CC BY 4.0	https://doi.org/10.5281/zenodo.13227905
Enclosure_modifications	Zip	CC BY 4.0	https://doi.org/10.5281/zenodo.13227905
AprilTags_files	Zip	CC BY 4.0	https://doi.org/10.5281/zenodo.13227905
beecam2_preview	Python	CC BY 4.0	https://doi.org/10.5281/zenodo.13227905
queen_image	Img	CC BY 4.0	https://doi.org/10.5281/zenodo.13227905
worker01_image	Img	CC BY 4.0	https://doi.org/10.5281/zenodo.13227905
Software_setup	Docx	CC BY 4.0	https://doi.org/10.5281/zenodo.13227905
BeeCam_AprilTag_Manual	Docx	CC BY 4.0	https://doi.org/10.5281/zenodo.13227905
Postprocessing	Zip	CC BY 4.0	https://doi.org/10.5281/zenodo.13227905

## 4 Bill of materials

**Table:** 

Designator	Component	Number	Cost per unit - currency	Total cost - currency	Source of materials	Material type
P1	Monocrystalline Solar Panel 195 W 12 V	1	$159.99USD	$159.99USD	Eco-Worthy	Other

P2	Lithium Iron Phosphate Battery 100 Ah 12 V	1	$235.99USD	$235.99USD	Eco-Worthy	Other

P3	MPPT Solar Charger 40 A 12/24 V	1	$119.99USD	$119.99USD	Eco-Worthy	Other

P4	Waterproof Switch 12 V 20 A	6	$1.89USD	$11.34USD	Amazon	Other

P5	DC 12/24 V to 5 V USB-C Waterproof Converter	6	$6.50USD	$39.00USD	Eco-Worthy	Other

P6	Plastic Dustproof Waterproof IP65 Enclosure (5.9 × 5.9 × 3.5 in.)	6	$16.99USD	$101.94USD	Amazon	Other

P7	Raspberry Pi 4 Model B with 2 GB RAM	6	$45.00USD	$270.00USD	Mouser	Other

P8	Raspberry Pi Camera Module 3 Wide	6	$35.00USD	$210.00USD	Mouser	Other
P9	Real Time Clock PCF8523	1	$6.95USD	$6.95USD	Mouser	Other

P10	LED Strips 5 V 6.6FT IP65 Waterproof Natural White	1	$10.99USD	$10.99USD	Amazon	Other

P11	Panda PAU0D WiFi Adapter AC1200 USB with Dual Antenna	1	$27.99USD	$27.99USD	Amazon	Other

The bill of materials outlines the primary components of the system, designed for deployment in an apiary with six colonies, comprising one “queen” computer and five “worker” computers. We have included an Excel spreadsheet in our repository containing a more detailed bill of materials, listing every individual piece of hardware, along with links to websites for purchasing. We highly recommend referring to this file (Bill_of_Materials.xlsx).

## 5 Build instructions

*Assembling power module.*

1. Connect the cables from the battery (P2) to the solar charger controller (P3). Once the controller has been powered up, select the type of battery that is connected (“LiFePo4-4s” for our battery). Then, connect the solar panel (P1) to the controller. Pay special attention to the polarity of the wires: red cables correspond to positive voltage and black cables to ground.
2. Use six switches (P4) and join together all their POWER(+) cables. Connect their power cables to the LOAD(+) port of the controller (P3). Similarly, join the GROUND(−) cables of the switches together and connect them to the LOAD(−) port. Use electrical tape for waterproofing and electrically isolate the connections.
3. Cut portions of 18 gauge dual conductor electrical wire to reach from your solar controller with the switches to the entrances of your hives.
4. Peel both ends of the electrical wire to expose the red and black cables inside. For one end, connect the red cable to the LOAD(+) cable of the switch and the black cable to the switch GROUND(−) cable. Use the other end of the wire to connect the DC to USB-C block converter (P5) by matching the connection between cables with the same color. Red cables correspond to +12V DC power and black cables to ground. Make sure all connections are tight, waterproof, and isolated using electrical tape.
5. Use the USB-C male cable to supply power to each Raspberry Pi computer inside their enclosure by using the cable glands.

*Assembling enclosure and electronics.*

1. Start by modifying the waterproof IP65 enclosure (P6) as shown in Fig. 3. Print the file *enclosure_modifications.pdf* on translucent sticker paper, cut the edges, and place it on the bottom-center of the IP65 enclosure. Then, drill the corresponding circles with $3/32^{\prime }$, $13/64^{\prime }$ and $1/2^{\prime }$ drill bits. Using a 3/4 inch drill bit, make a hole on the top-left wall of the enclosure to fit the cable gland it comes with. Then, inside the enclosure, install the aluminum DIN rail using M3.5 × 6 mm Phillips bolts.
2. Assemble the Raspberry Pi (P7) by first connecting the camera (P8) cable to the camera port. Ensure the cable is folded properly and does not obstruct the CPU, as improper folding can generate noise from the CPU heat. Typically, glitches in the camera preview are due to incorrect cable folding or connection. For the “queen”, install the external clock PCF8523 (P9) using jumper wires connected to the corresponding GPIO pins. It is recommended to trim the jumper wire heads that attach to the GPIO pins, so the aluminum case fits correctly. Do the same for a pair of female-to-female jumper wires, connecting them to the GPIO pins for 5 V and GND. These wires will provide power to the LEDs later. Finally, install the aluminum heat sink case, making sure it presses tightly against the CPU with the thermal pads to ensure good heat dissipation.
3. Attach the Raspberry Pi camera (P8) to the enclosure using M2 bolts, nuts, and washers. Then, place the Raspberry Pi inside the enclosure and mount it over the DIN rail using two strips of double-sided Velcro. For the “queen”, install the WIFI dongle (P11) using the USB-C cable extension and the USB-A 3.0 to USB-C 90 degrees adapter, so it can fit inside the enclosure. Make sure to use the RPi’s USB 3.0 port to ensure high-speed WIFI connectivity.
4. Cut two pieces of LED strips (P10) and place them in the base of the enclosure as the printed sticker indicates. Then, solder male-to-male jumper wires to the 5 V and GND pads on both LED strips and connect them to the female-to-female jumpers previously connected to the RPi.
5. 3D printed entrances require the installation of a window for the camera to view the tunnel. Cut a piece of 3 × 2 in. of 1/32” clear polycarbonate. Then, use hot glue to attach it to the entrance window frame. Finally, use 8–32 × 3/4” screws and wing nuts to attach the entrance to the enclosure. Make sure to apply silicone waterproof sealant between the entrance and the enclosure to prevent moisture or dirt accumulation inside the entrance. Fig. 3 shows the hardware inside and outside the enclosure after all installations.

*Installing the operating system and software.*

Setting up the Raspberry Pi Operating System (OS) requires a Windows or Mac computer. Ensure your computer has internet access, then install the latest version of the Raspberry Pi Imager application. Next, download the image files “queen_image.img” and “worker01_image.img” from our repository. These files are “Bullseye” Raspberry Pi OS images that include all the necessary libraries, packages, scripts, and applications to run the queen and workers on a local wireless network. Detailed dependencies and configurations for these images are documented in the file “software_setup.dox”. Follow these steps to set up the OS and complete the additional configurations required.

1. Take a micro SD card and, using an adapter, connect it to the computer.
2. Launch the Raspberry Pi Imager application and select one of the image files (as a recommendation, start with the queen) as the Operating System. Then, initialize the installation.
3. Once the OS image has been installed correctly, you will notice the SD card has two partitions “root” and “boot”.
4. Properly eject the micro SD card from the computer and insert it in the Raspberry Pi with the correct enclosure, either for the queen or worker.
5. Connect a mouse, keyboard and monitor using two USB ports and the micro HDMI port correspondingly. Power up the RPi and the Raspbian OS will boot showing the graphical interface.
6. If the “queen” computer does not boot properly, the external clock needs to be updated following these steps:
  (a) Remove the coin battery from the external clock and remove the jumper wire that provides power.
  (b) Power up the RPi and select a WiFi network with internet access.
  (c) The RPi system time will automatically update from the WiFi connection. This may take a couple minutes. Insert the coin battery and connect the jumper wire with power back to the clock.
  (d) Execute the following terminal command to write the system time to the external clock *sudo hwclock -w*
  (e) To read the time on the external clock, execute the following terminal command *sudo hwclock -r*
  (f) If problems with the external clock persist, refer to the troubleshooting section of “BeeCam_AprilTag_Manual.dox” document in our repository.
7. Just for the “queen” computer, the WiFi dongle needs to be set up following these steps.
  (a) On the top-right corner of the desktop, right-click on the Network Adapter icon and select “advance options”/“edit connections”.
  (b) Select the WiFi network “queen” and click on the “gear” icon to access its configurations.
  (c) When you select the option “device” you will see two options for wlan1 and one option for wlan0. By default this connection has the wlan1 ID option pre-selected from the previous dongle we connected (for example 9C:EF:D5:F8:AC:A8).
  (d) Select the second option for wlan1 which corresponds to the new dongle you just connected (for example 9C:EF:D5:F8:96:99) and then click “save”. Your options will differ from the previous examples.
  (e) Reboot the RPi for the new settings to take effect. The WiFi network “queen” must always run automatically and be visible to other devices on the network.
8. Worker computers are differentiated by the last digit of their IP addresses and their hostnames. The static IPv4 addresses and hostnames must be manually configured for each worker computer after “worker01”. The following example is for “worker02” but the same procedure applies to all workers, up to “worker05” in our case.
  (a) Power up a Raspberry Pi with a micro SD card containing the OS image “workers_image.img”.
  (b) Open the terminal and enter the following command. *sudo raspi-config*
  (c) Navigate to “System Options” and then to “Hostname”
  (d) Enter the new hostname as “worker02”
  (e) Select “okay” then “Finish” and “yes” when prompted to reboot for the new hostname to take effect.
  (f) Open the terminal and enter the following commands to manually set the IPv4 address to 10.42.0.12 for “worker02”. *sudo nmcli c mod “queen” ipv4.addresses 10.42.0.12/24 ipv4.method manual sudo sudo con mod “queen” ipv4.dns 8.8.8.8*
  (g) Follow the same procedure for the rest of the workers while changing the last digit of the IP address. For example, the IPv4 address must be 10.42.0.13 for “worker03”

## 6 Operation instructions

Once the system hardware has been assembled and software installed, the system can be initialized by following these steps.

1. First, verify the power module is correctly functioning. Use the LCD screen of the solar charge controller to verify the solar panel voltage, the charging power, the battery voltage, etc. Use the solar charger manual to identify this information.
2. Once the power module operation has been verified, turn on the switches for the “queen” and “workers”.
3. Use your personal computer to access the WiFi networks available in the area. The network “queen” must be visible. Select the network and use the password: *queen1234* to access it.
4. Once the WiFi connection with the network “queen” has been established, launch the application RealVNC viewer. You must have previously installed this application on your personal computer.
5. In the top bar of the application, type the static IP address for the “queen”: 10.42.0.1
6. A small window will pop up. There, you must type the RPi computer username and password. For the “queen” the username is *master* and the password is *1234*
7. After you click “Ok”, the “queen” desktop will be shown. By default the queen and workers launch the executable “BecamPreview” when booting. If the camera preview is not displayed, in the top-left corner you will find the executable “BeecamPreview”. Double-click on it, and a small window will pop up. Select the option “Execute”. A window with the camera view should be visible after a few seconds. This means the program is running and the “queen” computer is ready to collect data. Fig. 5(a) shows the program execution process.
8. To access the workers, the procedure is similar. In the main RealVNC viewer window, type the IP address for the “worker” in the top bar. For example for “worker01” type 10.42.0.11
9. For all the “workers” the username is *pi* and the password is *1234*
10. After you click “Ok”, the “worker” desktop will be displayed with the camera view of the entrance, meaning the worker is ready for collecting data.
11. Connect to each worker and verify that the camera previews are running on the desktop.
12. Once all connections to the “queen” and “workers” have been established and verified, it means the system is correctly working and collecting data.
13. Printed tags can be placed in front of the camera and the preview should detect the tags by drawing a blue square around their border with the ID number printed next to it. Information about detections can be found in the folder “Documents”/“Data” in the “queen” computer.
14. To transfer data, right-click on the “RealVNC” icon on the top-right corner of the desktop. Then select “File Transfer” and click on “send files”. Choose the path “Documents”/“Data” and select the files you want to transfer. More detailed steps can be found in the file “BeeCam_AprilTag_Manual.dox”.

## 7 Validation and characterization

Six BeeCam-AprilTag systems were built and installed at three different locations in Pennsylvania and three in New York (USA) from March to July 2024 as part of a multidisciplinary research project that aims to study and estimate the maximum and most common distances that honey bees travel in organic-farm context by tagging newly-emerged bees. Our system is one of the tools the project targets to use to estimate the trip duration trends among colonies in similar context. Further analysis and calibration experiments are part of the project that in future publications aim to use trip duration data to estimate the foraging distances traveled by honey bees. Therefore, as part of our initial experiment, a total of 7000 unique tags were used for the season to identify individual honey bees across six different colonies per apiary. Tag ID numbers were allocated to colonies based on the highest digit. For example, the ID numbers 1000–1999 were allocated to the colony monitored by “worker01”. The ID numbers 2000–2999 were allocated to the “worker02” colony, and so on for each worker. The ID numbers 6000–6999 were allocated to the “queen” colony. Following this procedure, honey bees were tagged approximately every two weeks using 100 tags per colony in consecutive order. For each tagging session, the date, ID numbers used, and other important details were recorded. For the first two weeks, we used tags between 0–999 to test the system. Then we began using the protocol described previously.

The data for each apiary were collected from the field and uploaded to the cloud every two weeks. In order to analyze the data and extract trips from detections, we developed a post-processing code called “analysis_colony.py”. This Python script processes the data files for each colony by first extracting detections in chronological order. These detections are then grouped into events based on their temporal proximity. Two consecutive detections must occur within a 60-s window to be grouped as the same event; if the next detection occurs after this 60-s interval, it is treated as the start of a new event. This approach ensures that the minimum trip duration detected by our system is one minute, helping to differentiate between brief exits and entries. The events are categorized by the algorithm as “enter”, “exit”, or “unknown”. After this initial grouping, the algorithm sorts all events chronologically for each ID. A trip is defined as an exit event followed by a consecutive entry event. The code then filters out any non-consecutive exit and entry events, discarding failed exits, failed entries, and unknown events to ensure only valid trips are retained in the final data set. Once all trips are found, the script generates a report containing statistical analysis of the detections, events, and trips for the colony. It also generates histograms and a spreadsheet titled “Events_by_ID.xls” that contains all identified events.

**Fig. 6:**
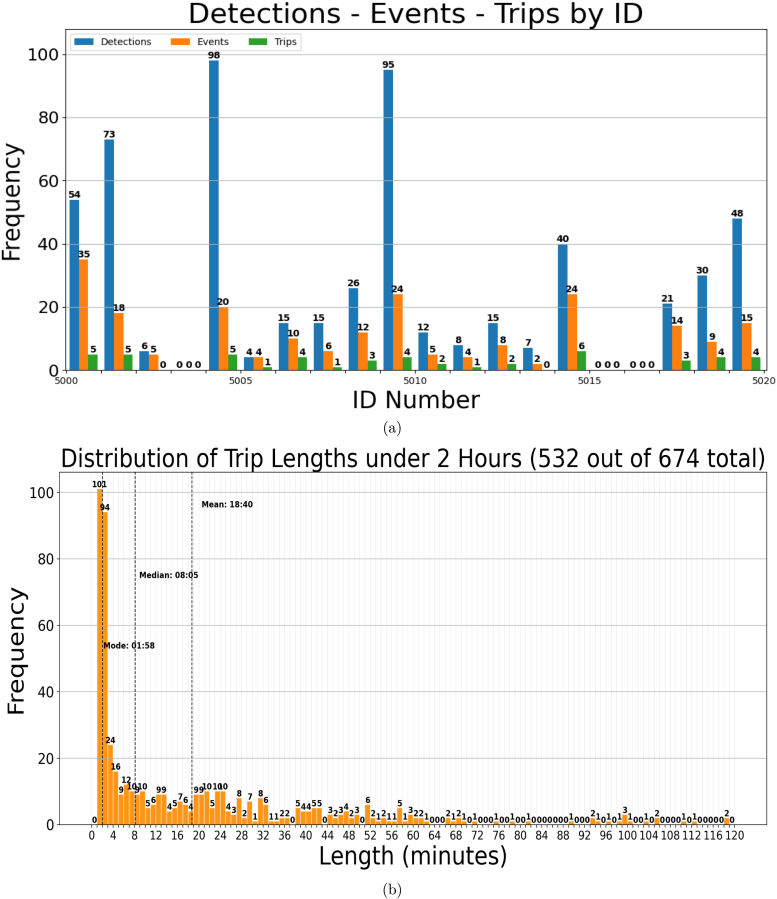
Post-processing plots. (a) Histogram of Detections-Events-Trips per ID number. (b) Histogram of number of trips per duration range in minutes.

As an example, we present the post-processing results for the apiary located at Spring Mills, Pennsylvania for week “2xWeek02” and “worker05” for ID numbers between 500 to 599 and 5000 to 5099. Fig. 6(a) shows the histogram for detections, events and trips for ID numbers between 5000 to 5020, meanwhile Fig. 6(b) shows the distribution of trip duration and the statistical parameters like mean, median, and mode. Over a course of 21 days of data collection the system generated 15,736 detections, obtaining 3297 events with an average of 4.27 detections per event. 32% were categorized as “enter” events, 18.77% as “exit” events and 49.23% as “unknown”. The system was able to identify 675 trips, where 533 trips were less than 2 h. The distribution of trip lengths leans heavily towards the 1–4 min range, with mode of 1 min and 58 s and a mean of 18 min and 38 s. More statistical parameters and plots can be found in the folder “Post-processing-example.zip”.

Our results indicate that most flights duration fall within the 1–4 min range, which align with two key factors we expected. First, one of our main Refs. [19], which describes a system similar to ours, reported trip duration estimates that correspond closely with our findings, showing a similar distribution of flight times. Second, based on the specific landscape of our experimental site — surrounded by abundant floral resources — our entomologist collaborators anticipated shorter trips. Additionally, it is important to note that honey bees leave the hive for many reasons beyond foraging. For instance, they often make short trips, known as “cleansing flights”, to defecate outside the hive, which typically last only a few seconds to a minute. On cloudy days, bees may also briefly exit the hive to check weather conditions before deciding whether to forage. In addition, new foragers frequently engage in “orientation flights” to familiarize themselves with the landscape and visual cues necessary for homing. These orientation flights generally last about 250–300 s [31]. All these specific behaviors results in high activity levels at the colony entrance, making detection challenging. During field experiments, we observed that bees often move quickly through the entrance, especially on warm, sunny days, which can lead to missed detections due to their speed. Additionally, bees sometimes crawl upside down through the entrance, obscuring the tags from the camera. This was a common behavior that affected detection rates. Another challenge was maintaining the clarity of the camera’s view through the translucent glass, which often became dirty due to pollen carried by bees or debris, including dead bees, obstructing the entrance. Our beekeeper collaborators cleaned the entrances every 10–15 days to ensure clear visibility for the cameras. However, it was not feasible to control bee behavior to ensure perfect visibility of the AprilTags at all times. As a result, the system registered a significant number of unknown events, which were likely caused by erratic bee behavior or simply bees lingering near the entrance

**Fig. 7:**
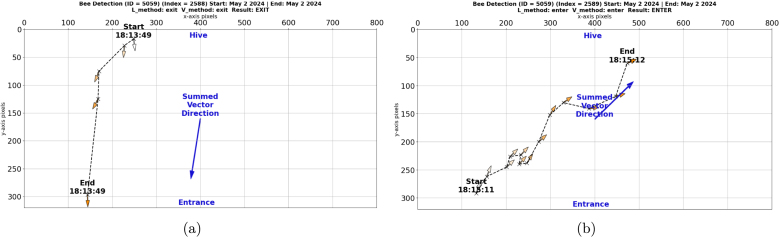
Detections plot per event for trips produce by a worker honey bee with ID 5059. (a) Detections when tag ID 5059 exited the hive. (b) Detections when tag ID 5059 entered the hive.

In order to further analyze the process of trip identification, we developed a script called “Events_plot.py”. This program plots each detection in an event as a vector within the camera’s field of view (800 × 320 pixels). Fig. 7 shows the identification of a trip produced by a honey bee carrying an AprilTag with ID number 5059. Fig. 7(a) drafts the trajectory produced by the detections of the honey bee exiting the hive by walking through the entrance. Fig. 7(b) drafts the trajectory of detections when the honey bee enters the hive after one minute and 23 s. Our goal is to improve the post-processing algorithm and experimental setup to increase the number of identified trips and reduce the unknown events. In a future publication, we will provide a detailed description of this algorithm along with statistical analysis comparing experimental results among colonies. For the latest version of the post-processing code refer to our GitHub repository: https://github.com/AERS-Lab/BeeCam-AprilTag.

**Fig. 8:**
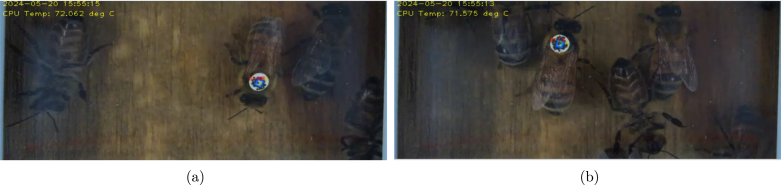
Screenshoot of beecam2_preview program. (a) Honey bee detected when it exits the hive. (b) Honey bee detected when it enters the hive.

In summary, this work presented the BeeCam-AprilTag system, an autonomous imaging system capable of monitoring in-and-out activity at the entrances of honey bee hives by attaching AprilTags to thousands of bees over several weeks. The system was successfully deployed for long-term monitoring and used to determine the trip lengths of identified honey bees. Our modular design proved to be robust against harsh environmental conditions and simple to repair in case parts needed to be replaced. Basing our system on Raspberry Pi hardware allowed us to access a variety of open-source tools and documentation, facilitating the connection of peripherals. This versatility can be further utilized to add more sensors to our current design, expanding the collection of relevant information about the colony and external stressors that may affect it. This contribution to entomology can also be applied to the study of other types of insects or any area of biology that requires low-cost, open-source instrumentation for long-term outdoor deployment (see Fig. 8).

## CRediT authorship contribution statement

**Diego Penaloza-Aponte:** Writing – original draft, Visualization, Software, Resources, Methodology, Formal analysis, Conceptualization. **Sarabeth Brandt:** Writing – review & editing, Software, Methodology, Formal analysis, Data curation. **Erin Dent:** Software, Formal analysis. **Robyn M. Underwood:** Validation, Resources, Project administration, Investigation, Funding acquisition, Conceptualization. **Benedict DeMoras:** Validation, Resources, Investigation. **Selina Bruckner:** Validation, Resources, Investigation. **Margarita M. López-Uribe:** Writing – review & editing, Supervision, Funding acquisition. **Julio V. Urbina:** Writing – review & editing, Supervision, Funding acquisition.

## Declaration of competing interest

The authors declare that they have no known competing financial interests or personal relationships that could have appeared to influence the work reported in this paper.
